# Differences in psychiatric care utilisation among unaccompanied refugee minors, accompanied migrant minors, and Swedish-born minors

**DOI:** 10.1007/s00127-020-01883-z

**Published:** 2020-05-13

**Authors:** Lovisa Axelsson, Sofie Bäärnhielm, Christina Dalman, Anna-Clara Hollander

**Affiliations:** 1 Stockholm, Sweden; 2 Center for Psychiatric Research, Department of Clinical Neuroscience, Karolinska Institutet (KI), Stockholm, Sweden; 3 Transcultural Centre, Region Stockholm, Stockholm, Sweden; 4 Epidemiology of Psychiatric Conditions, Substance Use and Social Environment (EPiCSS), Department of Global Public Health Sciences, KI, Solnavägen 1E, 171 77 Stockholm, Sweden; 5 Centre for Epidemiology and Community Medicine, Region Stockholm, Stockholm, Sweden

**Keywords:** Refugee, Unaccompanied refugee minor, Utilisation of psychiatric care, Population study

## Abstract

**Purpose:**

To better understand underutilisation of psychiatric care among migrant children, we compared utilisation of psychiatric care among unaccompanied refugee minors and accompanied migrant minors, with Swedish-born minors.

**Methods:**

Using a large longitudinal database of linked national registers, we established a retrospective cohort of 1,328,397 people born 1984–1988 comparing minors born in Sweden to 2 Swedish-born parents (95.4%) to minors who had been arriving in Sweden between 2002 and 2011 with a permanent resident permit and were either unaccompanied refugee minors (0.4%), or accompanied migrant minors (4.0%). The outcome measures were different measures of psychiatric care including in- and outpatient care, and prescribed psychotropic medication.

**Result:**

Compared with the Swedish-born minors the unaccompanied refugee minors had a higher likelihood of utilisation of all psychiatric care except ADHD medication. However, compared with accompanied migrant minors, the Swedish-born minors had a higher likelihood of having utilised psychiatric care.

**Conclusion:**

Our study shows that during the first years of living in Sweden, there seems to be fewer barriers to psychiatric care for unaccompanied refugee minors compared to the accompanied migrant minors. There are a number of possible reasons for this including stronger ties with the Swedish society.

## Background

The United Nations High Commissioner for Refugees (UNHCR) has documented the highest ever number of people who are forced to leave their homes at over 70.8 million people [1, 2]. Of these, about 25.9 million are classified as refugees in accordance with the United Nations Refugee Convention. Conferring to the UN Conventions, all persons under 18 years are considered to be children, and approximately half of the world’s refugees are children. Significant portions of these children have fled without parents, guardians or other relatives [2] and are known as unaccompanied refugee minors. Refugee children who migrate in the company of at least one parent or guardian, or who have at least one parent or guardian who is already resident in the new country will henceforth in this article be called accompanied minors.

All refugee children are at risk of having been exposed to stressful experiences before, during and post-migration [3]. Studies of refugee children have shown that exposure to violence is a risk factor for mental illness, whereas stable settlement and social support in the host country has a positive effect on the refugee child’s psychological functioning [3]. Unaccompanied refugee minors are in an even more vulnerable position than other refugee minors due lack of a guardian during flight and in the host country [4]. Refugee children in high-income countries are at increased risk of mental illness as compared with children of the general population [3]. Among refugee children, several European studies have shown that common mental disorders (referring to anxiety, depression, obsessive–compulsive disorder and post-traumatic stress disorder (PTSD)) are more common in unaccompanied refugee minors than accompanied minors [4–7].

Upon arrival to Sweden, all unaccompanied refugee minors are provided with a custodian who is “responsible for the child’s personal relationships and manage its affairs”, in line with Swedish Law [8]. During the time of the study, unaccompanied refugee minors were usually placed within the care of an institution or in foster care. Accompanied minors (both refugees and non-refugees) live with their parents or guardian in housing arranged by the parents or by the municipality [8]. This means that during their first years in Sweden, unaccompanied refugee minors are in closer contact with the Swedish social system through the staff at the institutions or foster parents, compared with accompanied minors.

A number of studies have shown that migrants utilise less psychiatric care, including in- and outpatient care, and prescribed and purchased psychotropic drugs, compared to the general population, but the duration of residence in the country is associated with increased psychiatric care utilisation [9–12]. These findings have generated the hypotheses that closer ties with the social system in the host country increase the likelihood of psychiatric healthcare utilisation among migrants. A hypothesis that so far has not been explored. Comparing unaccompanied refugee minors, accompanied minors with Swedish-born minors can shed further light on this question. We hypothesise that due to the elevated risk of mental illness and closer ties with the Swedish social system unaccompanied refugee minors will be the most likely among these three groups to utilise psychiatric care. We also hypothesise that due to a lower risk of mental illness, but stronger ties with the Swedish social system, Swedish-born minors will have intermediate utilisation of psychiatric care, and that, despite high risk of mental illness, but due to weak ties with the Swedish social system, the accompanied minors would have the lowest likelihood of utilising psychiatric care. In relation to time to first contact with psychiatric care since immigration, we also hypothesise that unaccompanied refugee minor would have shorter times to psychiatric care than accompanied minors.

### Aims of the study

The first aim of this study is to explore differences in utilisation of psychiatric care between unaccompanied refugee minors and accompanied minors from the same country of origin during their first years in Sweden and further to compare the two groups with Swedish-born minors. The second aim is to test if there is a difference in time from immigration to first contact with psychiatric care for the unaccompanied refugee minors as compared with the accompanied minors.

## Methods

### Study design and population

We established an open retrospective cohort of 1,328,397 people born 1984–1988 followed from January 1st 2002 to December 31st 2011. The population was divided into three groups. The first was persons born in Sweden to two Swedish-born parents (*n* = 1,267,938; 95.4%). The second and third group had arrived in Sweden between 2002 and 2011 with a permanent resident permit and were either unaccompanied refugee minors (*n* = 6133; 0.4%) or accompanied minors (*n* = 54,326; 4.0%).

To permit valid comparisons between unaccompanied refugee minors and accompanied minors, we restricted the sample to minors arriving from the same countries of origin. The accompanied minors were included regardless of if they or their parents were considered refugees or not. We excluded people without an official residence permit in Sweden—that is, undocumented migrants or people with an official asylum decision pending. Due to missing or incorrect data, we excluded 1125 persons (0.08%). In the Cox-regression analysis, we excluded additional 1957 (0.14%) persons due to incorrect data on survival times.

### Data sources

We extracted data from a large, longitudinal database of linked national registers, known as Psychiatry Sweden including all people officially resident in Sweden after 1 January 1932, linked via a unique personal identity number and anonymized by Statistics Sweden for research purposes. We obtained relevant outcome, exposure, and covariate data from this linkage. From Statistics Sweden we obtained: the register of the total population (RTB), to identify cohort participants and obtain basic demographic data (birth date, sex, country of birth); the multi-generation register to link participants to their parents for identification of the Swedish-born population; the register on immigration and emigration data (STATIV) to obtain migration and refugee data, including the unaccompanied refugee minors. From the National Board of Health and Welfare, we used the National Patient Register for data on psychiatry (inpatient care since 1973 and outpatient care since 2006) the Prescribed drug registry for data on prescribed and purchased drugs (since July 2005) the Cause of Death Register for causes of death (since 1951).

### Outcomes

Psychiatric care use was studied as a combined variable (called any psychiatric care) and defined as receipt of the first time use of any psychiatric services during the study period and coded as binary. Psychiatric care use was also studied in separated: inpatient care (use of hospital psychiatric services), outpatient care (specialist care in community psychiatric services) and prescribed and purchased psychotropic medication including psychotropic prescriptions were categorised into Antidepressants (Anatomic Therapeutic Chemical [ATC] code N06A), ADHD medications (ATC N06BA01 to NO6BA04, N06BA09), Antipsychotics (ATC N05A), Anxiolytics (ATC N05A) and Sedatives/Hypnotics (ATC N05C). The type of care is not diagnose specific, however, inpatient care is care given for patients presenting with severe mental health and in need of comprehensive support and this type of care is nowadays relatively rare. The prescriptions include those from general practitioners and specialists in outpatient settings, but not inpatient settings (0.2% of the population), as the latter is not recorded in the Swedish Prescribed Drug Register. All children in Sweden, including both unaccompanied refugee minors and accompanied minors are entitled to the same healthcare as Swedish-born minors. Sweden has a universal health insurance system with small out-of-pocket costs. During the time of the study, patients paid a maximum 2200 SEK (c. £220) per year for prescribed psychotropic drugs and the rest was paid by state subsidies [13].

The second outcome, for the migrant group only, was time between immigration to first psychiatric care, measured in years.

### Exposures

The primary exposure was minor status. Unaccompanied refugee minors were compared with accompanied minors and Swedish-born minors.

### Confounders

We included age, as a continuous variable, and sex as potential confounder.

### Statistical analyses

Demographic variables were compared using Chi-square tests. We reported basic descriptive statistics (Table 1). We fitted time-dependent Cox models to estimate hazard ratios (HR) for in- and outpatient care and psychotropic medication with 95% confidence intervals (95% CIs) (Table 2). Follow-up time used time in the cohort as underlying timescale adjusted for age and sex. Multivariate logistic regression was used to estimate odd ratios for specific psychotropic outcomes adjusted for age and sex (Table 3). Time to psychiatric care was compared using *T* tests. Statistical analysis was performed using SPSS and SAS.

### Sensitivity test of age

There are discussions of whether the age of unaccompanied refugee minors who immigrate close to age 18, is correct [14]. To test if misclassification of age among those close to 18 years old could influence the results we made a sensitive test. This was done by creating a subset excluding everybody aged 17 at immigration and running the analysis in this subset.

### Ethical approval

This research has ethical approval as part of Psychiatry Sweden “Psykisk ohälsa, psykiatrisk sjukdom: förekomst och etiologi,” approved by the Stockholm Regional Ethical Review Board (Number 2010/1185-31/5).

## Results

We established a retrospective cohort of 1,328,397, with Swedish-born minors (*n* = 1,267,938; 95.4%), unaccompanied refugee minors (*n* = 6133; 0.46%) and accompanied minors (*n* = 54,326; 4.0%) from the same country of origin, see Table 1. There was an even distribution of boys and girls among the Swedish-born and accompanied minors, but in the unaccompanied refugee minor group, there were 80% boys, see Table 1. The mean age at end of study was highest among the Swedish-born minors, and the unaccompanied refugee minors were older at time of immigration compared accompanied minors. For more details about the population, see Table 1. When compared with the Swedish-born minors, a larger proportion of the unaccompanied refugee minors had utilised any form of psychiatric care except for ADHD medication, neuroleptics and antidepressants (see Table 1). Whereas, when compared with accompanied minors, a larger share of the Swedish-born minors had experienced all outcomes (see Table 1).

**Table 1:** Population characteristics by exposure, *N* and (%)

	Unaccompanied refugee minors	Accompanied minors	Swedish-born minors	Total
*N* (%)	6133 (0.46)	54,326 (4.01)	1,267,938 (95.4)	1,328,397
Boys	4909 (80)	28,465 (52.4)	651,393 (51.4)	684,767 (51.5)
Mean age at immigration	15.7 (SD = 1.51)	12.37 (SD = 3.41)	−	12.71 (SD = 3.42)
Mean age at end of study	18.33 (SD = 2.66)	17.69 (SD = 3.38)	20.19 (SD = 4.11)	20.08 (SD = 4.12)
Region of origin				
West Africa	55 (0.9)	1247 (2.3)		
Africa Other	159 (2.6)	2439 (4.5)		
Central Asia	2782 (45.4)	4986 (9.2)		
Northeast Asia	12 (0.2)	1600 (2.9)		
Southeast Asia	20 (0.3)	6210 (11.4)		
Russia and the Baltic states	21 (0.3)	2776 (5.1)		
Former Yugoslavia	55 (0.9)	4846 (8.9)		
Iraq	993 (16.2)	14,758 (27.2)		
Iran	109 (1.8)	1726 (3.2)		
Middle east	124 (2.0)	4380 (8.1)		
North Africa	28 (0.5)	528 (1.0)		
Central America	2 (0.03)	571 (1.1)		
Unknown	36 (0.6)	76 (0.1)		
Somalia-Eritrea-Ethiopia-Djibouti	1733 (28.3)	6568 (12.1)		
South America	4 (0.1)	1615 (3)		
Total	6133 (100.0)	54,326 (100.0)		
Inpatient care	239 (3.9)	967 (1.8)	46,088 (3.6)	47,294 (3.9)
Outpatient care	745 (12.1)	3509 (6.5)	148,476 (11.7)	152,730 (11.5)
Prescribed psychotropic drugs	1340 (21.8)	3824 (7.0)	196,055 (15.5)	201,219 (15.1)
ADHD medication	14 (0.2)	282 (0.5)	35,864 (2.8)	36,160 (2.7)
Tranquilisers	668 (10.9)	2073 (3.8)	101,306 (8.0)	104,047 (7.8)
Neuroleptics	94 (1.5)	425 (0.8)	20,147 (1.6)	20,666 (1.6)
Antidepressants	531 (8.7)	1502 (2.8)	116,619 (9.2)	118,652 (8.9)
Sedatives	758 (12.4)	1443 (2.7)	75,444 (6.0)	77,645 (5.8)

The crude hazard ratios in Table 2 showed that prescribed psychotropic drugs were higher for unaccompanied refugee minors, compared with Swedish-born minors, and accompanied minors (see Table 2 and 3). When adjusted for age and sex unaccompanied refugee minors had a higher risk of utilisation of in- and outpatient care and prescribed psychotropic drugs compared with Swedish-born minors, and accompanied minors (see Table 2). When studying the prescribed psychotropics in detail (Table 3), it showed that unaccompanied minors had higher odds of utilising all prescribed psychotropic drugs, except for ADHD medication, compared to Swedish-born minors and accompanied minors (see Table 3).

**Table 2:** Hazard ratios (HR) and 95% confidence interval (CI) for the (95% CIs) of the outcomes inpatient care, outpatient care and total prescribed of psychotropic drugs, by exposure

OR	Inpatient care		Outpatient care		Prescribed psychotropic drugs	
Model	1*	2**	1*	2**	1*	2**
Swedish-born minors as reference						
Unaccompanied refugee minors	0.99 (0.80–1.10)	1.30 (1.10–1.45)	1.01 (0.94–1.10)	1.10 (1.01–1.18)	1.74 (1.64–1.83)	1.10 (1.01–1.19)
Accompanied migrants minors	0.42 (0.40–0.05)	0.57 (0.54–0.61)	0.55 (0.53–0.57)	0.58 (0.56–0.60)	0.48 (0.46–0.50)	0.58 (0.56–0.60)
Girls	–	1.13 (1.12–1.14)	–	1.13 (1.12–1.14)	–	1.12 (1.12–1.14)
Age	–	0.98 (0.98–0.98)	–	0.98 (0.98–0.98)	–	0.98 (0.98–0.98)

*Model 1: crude

**Model 2: adjusted for gender and age at the end of the study

**Table 3:** Hazard ratios (HR) and 95% confidence interval (95% CIs) of the outcome prescribed psychotropic drugs in detail, by exposure

OR	Prescribed psychotropic drugs in detail										
	ADHD medication			Tranquilisers		Neuroleptics		Antidepressants		Sedatives	
Model	1*		2**	1*	2**	1*	2**	1*	2**	1*	2**
Swedish-born minors as reference											
Unaccompanied refugee minors	0.08 (0.05–0.13)	0.06 (0.04–0.10)		1.41 (1.30–1.53)	2.21 (2.04–2.40)	0.96 (0.79–1.18)	1.27 (1.04–1.56)	0.94 (0.86–1.02)	1.58 (1.44–1.73)	2.23 (2.07–2.41)	3.25 (3.01–3.51)
Accompanied migrants minors	0.18 (0.16–0.20)	0.14 (0.13–0.16)		0.46 (0.44–0.48)	0.62 (0.59–0.65)	0.49 (0.44–0.54)	0.65 (0.59–0.71)	0.28 (0.27–0.30)	0.41 (0.38–0.43)	0.43 (0.41–0.46)	0.59 (0.56–0.62)
Girls	–	0.57 (0.56–0.58)		–	1.87 (1.84–1.89)	–	1.14 (1.11–1.18)	–	1.94 (1.92–1.97)	–	1.44 (1.42–1.46)
Age	–	0.91 (0.90–0.91)		–	1.12 (1.12–1.12)	–	1.11 (1.10–1.11)	–	1.15 (1.15–1.15)	–	1.12 (1.12–1.12)

*Model 1: crude

**Model 2: adjusted for gender and age at the end of the study

We found significant differences in time from immigration to first utilisation of psychiatric care for the unaccompanied refugee minors when compared with accompanied minors. The mean time from immigration to inpatient psychiatric care for unaccompanied refugee minors was 1.58 years [Standard deviation (SD) = 1.75], whereas the time for accompanied minors was 3.86 years (SD = 2.42), this difference was statically significant (*p* < 0.001). The mean time from immigration to outpatient care for unaccompanied refugee minors 1.37 years (SD = 1.64) years and for accompanied minors 3.10 years, (SD = 2.44) (*p* < 0.001). The difference in time between the two groups in relation to first prescribed psychotropic drugs was also significantly different (*p* < 0.001), for unaccompanied refugee minors, time to first prescribed psychotropic drug was 1.58 years (SD = 1.79) compared to accompanied minors who had a slightly longer duration of 4.47 years (SD = 2.71).

### Sensitivity test of age

To test if misclassification of age among those who immigrated close to age 18 could influence the results, we made a sensitivity analysis by excluding everybody aged 17 at immigration. This test excluded 1954 unaccompanied refugee minors and 6428 accompanied minors. The results of the logistic regression did not differ significantly when excluding the 17 years old at immigration, thus we decided against the exclusion of this group in the main analysis.

## Discussion

We first hypothesised that due to the elevated risk of mental illness and closer ties with the Swedish social system, unaccompanied refugee minors would be more likely to utilise psychiatric care compared to Swedish-born minors and accompanied minors. This hypothesis was supported with an exception of ADHD-medication use, for which Swedish-born minors had a higher use. We also hypothesised that accompanied minors, despite their higher risk of mental illness, would have a lower utilisation of psychiatric care, due to weaker ties with the Swedish social system. This hypothesis is also supported with the findings from the present study. In addition, the hypothesis regarding waiting times was supported as unaccompanied refugee minors had significantly shorter waiting times than the accompanied minors.

The stark differences between unaccompanied refugee minors and accompanied minors cannot be explained with the protective factor of migrating with parents or guardian alone, as accompanied minors had a significantly lower utilisation than Swedish-born minors who also had the support of parents, but on average, lack the additional risk factors of accompanied minors. The higher likelihood of utilising all psychiatric care including prescribed psychotropic drugs (except ADHD medication) among unaccompanied refugee minors is expected as several European studies have shown that unaccompanied refugee minors have a high risk of mental disorders such as anxiety, depression PTSD compared to both accompanied and the minors born in the host country [4–7]. Longitudinal research of mental illness among unaccompanied refugee minors has shown that the mental illness is not disappearing as time goes by [4, 7]. The higher risk of mental ill health among unaccompanied refugee minors is paired with this group also having an easier and quicker access to Swedish psychiatric healthcare as they are by law provided with a custodian who is “responsible for the child’s personal relationships and manage its affairs”. In 1997, Jorm et al. coined the term “mental health literacy” to refer to the “knowledge and beliefs about mental disorders which aid their recognition, management or prevention” (p. 182) [15]. Novelty of the health system for the family, and low language proficiency contributes to lower mental health literacy among migrants than the native-born population. However, the custodian assigned to the unaccompanied refugee minors is familiar with the Swedish social system and the Swedish healthcare system, and therefore, have health literacy in Sweden. There are also various forms of accommodation for unaccompanied refugee minors, for instance, in institution or in foster care, with staff or foster parents to help with navigation within the Swedish healthcare system and who could assist in terms of transcultural guidance. The contact with the healthcare system for accompanied minors is dependent on their parents or guardians who are equally as new to the Swedish healthcare system as the migrant minor. Studies have shown barriers to mental healthcare exist in many migrant groups [16, 17] also among refugees to Sweden [18]. Studies have also shown that some migrant groups prefer to resolve mental health problems within the family and not seeking help [19]. This strategy of resolving mental health problems could be problematic in times of forced migration, where the family network is lacking thus making this strategy much weaker than intended.

Stigma and prejudice provide an additional explanation for the differences between unaccompanied refugee minors and accompanied minors. In many settings, mental illness is still associated with stigma and prejudice, however, these two social processes are more pronounced and repressing in some areas of the world, and in certain cultures and contexts. Both stigma and prejudice can affect an individual’s pathway to utilisation of psychiatric care [20]. As unaccompanied refugee minors are under the supervision of staff trained in a Swedish setting, where mental illness is attached with less stigma, there might be less of a barrier for this group to seek care when compared with the accompanied minor group. Unaccompanied refugee minors have closer ties with the Swedish social system, therefore, offering a possible explanation to the difference in time to first treatment. A potential alternative reason to the difference in help seeking between unaccompanied refugee minors and the accompanied migrants could be related to differences in mental health care in countries of origin. This explanation is not likely, as both groups came from the same countries of origin. Even though, there are diverse health care systems and cultural traditions in countries of origin all come from countries with underdeveloped and war affected health care systems [21–23].

Unaccompanied refugee minors and accompanied minors all had a higher likelihood than Swedish-born minors to have utilised any form of psychiatric care, except for ADHD medication. This is in line with two Swedish studies that found that utilisation of ADHD medication was lower among migrant minors in general [9, 10] as compared to Swedish-born minors, and their odds of not utilising medication increased as the share of migrants in the area of living increased [10]. Patients’ explanatory models of their symptoms can differ markedly from those of the treating clinician, and this difference can profoundly influence utilisation and possibly the chance of referrals. For mental health professionals, different cultural variation in presentation of psychiatric symptoms contributes to risk of being misdiagnosed [24]. Transcultural awareness among staff can bridge cultural differences between patients and care and if this is low, it might be difficult for staff to diagnose.

In a review of mental health literacy interventions, the authors conclude that the barriers to psychiatric healthcare include, stigma of attending mental health services or receiving a psychiatric diagnostic label; limited knowledge of the types of services available; lack of availability in culturally sensitive services; and language barriers [25]. Additionally, the authors stress that many studies have shown that these barriers could be addressed by mental health literacy interventions if these interventions are culturally sensitive taking cultural diversity into account.

## Strengths and weaknesses

This study has several methodological strengths. It was based on a large, national population-based cohort of more than 1.3 million people using linked Swedish register data. Swedish register data are known to be reliable for research purposes [26]. This register is highly complete, recording all psychiatric contacts from inpatient settings from 1987 onwards and from outpatient settings since 2001 and all prescribed and purchased psychiatric medications since 2005. Sensitivity analyses suggested that our results were not due to misclassification of birth year.

We have no data to compare actual psychiatric care needs with utilisation. To study if there is true underutilisation, it would be important to compare with actual needs of these groups. However, this study still gives valuable information about barriers to care. Despite Sweden has a universal health insurance system with small out-of-pocket costs, these costs could be considered burdensome for the less affluent groups of society. We only have data on prescribed psychotropic drugs for the last years of the study (second half of 2005) and the prescribed psychotropic drugs only included those prescribed by general practitioners and specialists in outpatient settings, but not in inpatient settings (0.2% of the population), as the latter are not recorded in the Swedish Prescribed Drug Register. Thought this may have led to slight under-ascertainment, we have no reason to believe that this would have introduced differential bias.

Studies show that some psychiatric condition receive care quicker than others, for instance psychotic disorders are found to have shorter time from onset to speciality care [27, 28]. Due to the unaccompanied refugee minors disrupted family environment resulting from forced separation with parents, this is the group might be at a higher risk of developing severe mental disorders [6]. As this study have no information of psychiatric disorders, the unaccompanied refugee minors seek psychiatric care for it could have been due to more severe psychopathology (severe depression, psychosis, bipolar disorder) and because of this, they had a faster and more intensive contact with psychiatry care.

## Implications

This study shows that although many migrant minors having low psychiatric care utilisation during the first years, this is not unavoidable as unaccompanied refugee minors have higher and faster utilisation of psychiatric care than accompanied minors have. These barriers are not only found among newly arrived migrants but also within the healthcare system itself, and further research is needed to explore these barriers. In addition, future research should focus on how to overcome these barriers, for instance having culturally sensitive interventions in place could be helpful and beneficial for migrant children. Another implication of the present study is that it provides a basis to better understand the effect of psychiatric care among migrant children. The severity of psychiatric morbidity could possibility explain unaccompanied refugee minors high levels of psychiatric care utilisation, however, it cannot explain accompanied minors lower levels of psychiatric care utilisation compared with Swedish-born minors, hence our interpretation that the closer ties with the Swedish social system helps unaccompanied refugee minors to receive adequate psychiatric care are still valid.

Our study shows that during the first years of living in Sweden, there seems to be fewer barriers to psychiatric care for unaccompanied refugee minors compared to accompanied minors. There are a number of possible reasons for this such as stronger ties with the Swedish social system among the unaccompanied refugee minors, low health literacy among the parents of accompanied minors, and low transcultural awareness among healthcare staff.
